# Expanded phenotypic spectrum of *FOXL2* Variant c.672_701dup revealed by whole-exome sequencing in a rare blepharophimosis, ptosis, and epicanthus inversus syndrome family

**DOI:** 10.1186/s12886-023-03189-5

**Published:** 2023-11-07

**Authors:** Zhi-Bo Lin, Zhen-Ji Chen, Hui Yang, Xing-Ru Ding, Jin Li, An-Peng Pan, Hai-Sen Sun, A.-Yong Yu, Shi-Hao Chen

**Affiliations:** 1 National Clinical Research Center for Ocular Diseases, Eye Hospital, Wenzhou Medical University, Wenzhou, 325027 China; 2 Oujiang Laboratory, Zhejiang Lab for Regenerative Medicine, Vision and Brain Health, Wenzhou, Zhejiang China; 3 Department of Ophthalmology, The Second Affiliated Hospital and Yuying Children’s Hospital of Wenzhou Medical University, Wenzhou, China; 4 Zhejiang Provincial Clinical Research Center for Pediatric Disease, The Second Affiliated Hospital of Wenzhou Medical University, Wenzhou, Zhejiang China

**Keywords:** *FOXL2*, Whole-exome sequencing, BPES, Anisometropia, Congenital cataracts

## Abstract

**Introduction:**

Blepharophimosis, ptosis, and epicanthus inversus syndrome (BPES) is a rare genetic disease with diverse ocular malformations. This study aimed to investigate the disease-causing gene in members of a BPES pedigree presenting with the rare features of anisometropia, unilateral pathologic myopia (PM), and congenital cataracts.

**Methods:**

The related BPES patients underwent a comprehensive ocular examination. Next, whole-exome sequencing (WES) was performed to screen for the disease-causing genetic variants. A step-wise variant filtering was performed to select candidate variants combined with the annotation of the variant's pathogenicity, which was assessed using several bioinformatic approaches. Co-segregation analysis and Sanger sequencing were then conducted to validate the candidate variant.

**Results:**

The variant c.672_701dup in *FOXL2* was identified to be the disease-causing variant in this rare BPES family. Combined with clinical manifestations, the two affected individuals were diagnosed with type II BPES.

**Conclusion:**

This study uncovered the variant c.672_701dup in *FOXL2* as a disease causal variant in a rare-presenting BPES family with anisometropia, unilateral pathogenic myopia, and/or congenital cataracts, thus expanding the phenotypic spectrum of *FOXL2*.

## Introduction

Blepharophimosis, ptosis, and epicanthus inversus syndrome (BPES; OMIM #110,100) is a rare, autosomally inherited disease that occurs in approximately 1 in 50,000 births worldwide [1]. BPES primarily affects the development of the eyes with four main characteristic features: eyelid dysplasia (telecanthus), small palpebral fissures (blepharophimosis), drooping eyelids (ptosis), and a tiny skin fold running inward and upward from the lower lid (epicanthus inversus) [2]. BPES can be further divided into two subtypes depending on the presence or absence of systemic involvement. In type I BPES, the eyelid abnormalities are co-inherited with premature ovarian failure (POF), while type II BPES only manifests as eyelid defects. In addition to the hallmark eye malformations, other common ocular signs include squint, nystagmus, microphthalmus, microcornea, and stenosis of the lacrymal canaliculi [3].

BPES is most commonly inherited in an autosomal dominant manner. Studies have revealed that *FOXL2* is the major disease-causing gene associated with BPES, accounting for 67% of cases [4, 5]. Other genes being reported to cause BPES with extended phenotypes include *KAT6B* [6], *SEPT9* [7], and *ITGB5* [8]. Due to the wide variety of BPES ocular manifestations, there is still a need to investigate the disease causal variants associated with the different ocular phenotypes of BPES. The BPES pedigree reported in this study was affiliated with the presence of anisometropia and unilateral pathologic myopia (PM). Moreover, the proband was diagnosed with congenital cataracts. Interestingly, BPES accompanied by congenital cataracts or PM has rarely been reported, and there is a lack of molecular genetic studies of BPES associated with anisometropia [9, 10].

Thus, this study investigated the disease-causing gene or variant in a family affected by a rare presentation of BPES using whole-exome sequencing (WES) with the aim of investigating the genotype–phenotype correlation of BPES with anisometropia, unilateral PM, and congenital cataracts.

## Methods

### Participants

Members of this family underwent comprehensive ocular examinations that included a best-corrected visual acuities (BCVAs) test, color face photography, corneal and conjunctival examination with a slit-lamp microscope, axial length examination with an IOL Master optical biometer, ocular ultrasound, and fundus examination. The distance between the inner canthus, the length and height of the eyelid fissure, and the muscle strength of the levator were also measured. The proband and his mother were diagnosed with BPES, however family members could not confirm the family history of BPES, thus this family was defined as a sporadic BPES pedigree. This study adhered to the tenets of the Declaration of Helsinki and was approved by the ethics committee of The Eye Hospital of Wenzhou Medical University. All participants signed written consent forms.

### Whole-exome Sequencing (WES) and Bioinformatics

Genomic DNA was extracted from peripheral blood samples obtained from each affected individual in this pedigree. WES was then performed using Illumina NovaSeq 6000. Average sequencing coverage was 100 × , and up to 95% of coverage was 20 × . The VeritaTrekker Variants Detection System was applied to detect the single-nucleotide variants (SNVs), insertions or deletions (InDels, < 50 bp), and copy number variants (CNVs, > 100 kb) in the whole exome region within 5 bp of splicing sites or within 50 bp of InDels. The raw data was analyzed and annotated by Enliven Data Annotation and Interpretation System.

### Variant assessment

In further analysis, synonymous SNVs (sSNVs) and non-coding region variants were excluded. Variants with a minor allele frequency (MAF) greater than 1% in 1000 Genome Project (1000G, ftp://1000genomes.ebi.ac.uk/vol1/ftp), Exome Aggregation Consortium (ExAC, http://exac.broadinstitute.org/), Genome Aggregation Database (gnomAD, https://gnomad.broadinstitute.org/), and dbSNP (http://www.ncbi.nlm.nih.gov/snp) were filtered out. The position with a reading depth less than 10 was filtered out with the aim of controlling the quality and reliability of the sequencing data. Estimation of potential deleteriousness among all candidate variants was determined using the following predictive tools: SIFT (http://sift.jcvi.org/), Polyphen-2 (http://genetics.bwh.harvard.edu/pph2/), MutationTaster (http://mutationtaster.org/), ClinVar (http://www.ncbi.nlm.nih.gov/clinvar), and CADD (https://cadd.gs.washington.edu/). The variants were categorized by pathogenicity – e.g., pathogenic, likely-pathogenic, uncertain significance, benign, and likely benign – according to the guidelines of the American College of Medical Genetics and Genomics (ACMG) [11], and the recommendations of CliGen Sequence Variant Interpretation (SVI) [12–14], combined with the database of Human Phenotype Ontology (HPO, https://hpo.jax.org/app/), Online Mendelian Inheritance in Man (OMIM, https://www.omim.org/), and Genetics Home Reference (GHR, http://www.ghr.nlm.nih.gov). The variant defined as "pathogenic" or "likely-pathogenic" will be regarded as a candidate disease causal variant and be included in further validation.

### Variant validation

To validate the variant identified by WES, Sanger sequencing was conducted. First, polymerase chain reaction (PCR) was performed to amplify the candidate disease causal variant region from genomic DNA with a pair of designed primers that covered > 50 bp upstream and downstream of the variant. The amplified products were sequenced by ABI 3500 Genetic Analyzer (Applied Biosystems, Carlsbad, California). After validation of the disease causal variant, co-segregation analysis was performed to confirm the variant being inherited from the proband’s affected mother but not the healthy father with a matching of the autosomal dominant inheritance mode. An online tool, SWISS-MODEL (https://swissmodel.expasy.org/interactive), was also applied to visualize the difference between the wild-type and variant protein structures.

## Results

### Clinical features

The 6-year-old proband’s (II:1, Fig. 1a) eyelids showed an epicanthus inversus. The distance between the two medial canthus was 33 mm, and the vertical and horizontal diameter of the palpebral fissure was 20 mm. The vertical diameter of the palpebral fissure was 3 mm, which was defined as severe bilateral ptosis. The myodynamia of the levator was 2 mm. Corneal and conjunctival examination of both eyes were within normal limits. Lenticular examination showed a posterior subcapsular opacity of the left eye lens only. A normal posterior segment in both eyes was observed by ocular ultrasound, with an axial length of 24.06 mm (OD) and 27.69 mm (OS). Fundus examination indicated lacquer cracks and peripapillary atrophy in the left eye but no abnormalities in the right eye (Fig. 2a). Spherical equivalent was 0.00D (OD) and -6.00D (OS). Unilateral amblyopia was diagnosed based on the poorly corrected vision in the left eye. After an ophthalmic examination, this patient underwent ptosis and epicanthus surgery in both eyes, as well as cataract surgery in the left eye (Fig. 2).

**Fig. 1:**
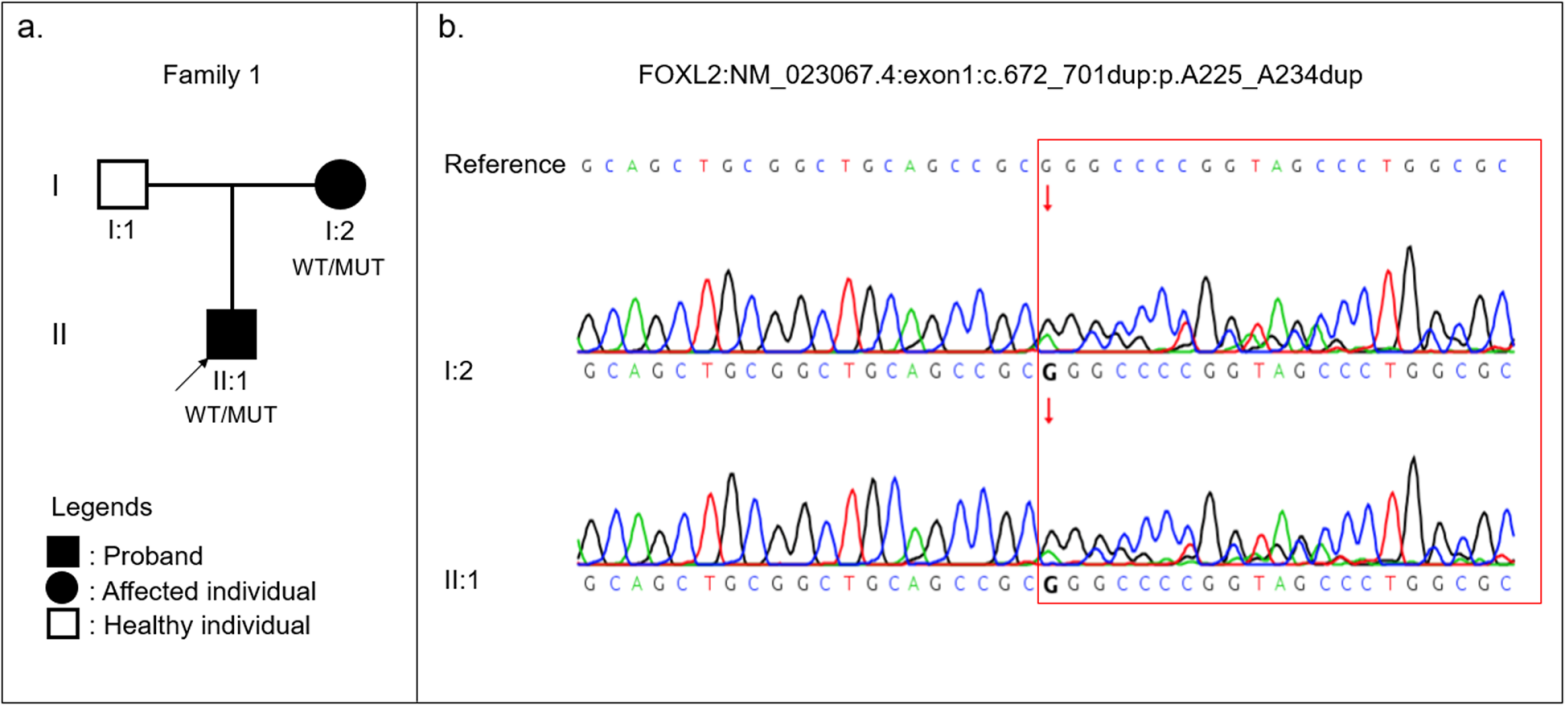
Pedigree and variants identified in a rare BPES family. **a** The pedigree shows the affected (II:1 and I:2) and unaffected (I:1) family members. Black arrow indicates the proband (II:1). **b** Red arrow refers to the start point of the inframeshift variant of both proband (II:1) and affected family member (I:2). Abbreviations: WT, wild-type; MUT, mutant

**Fig. 2:**
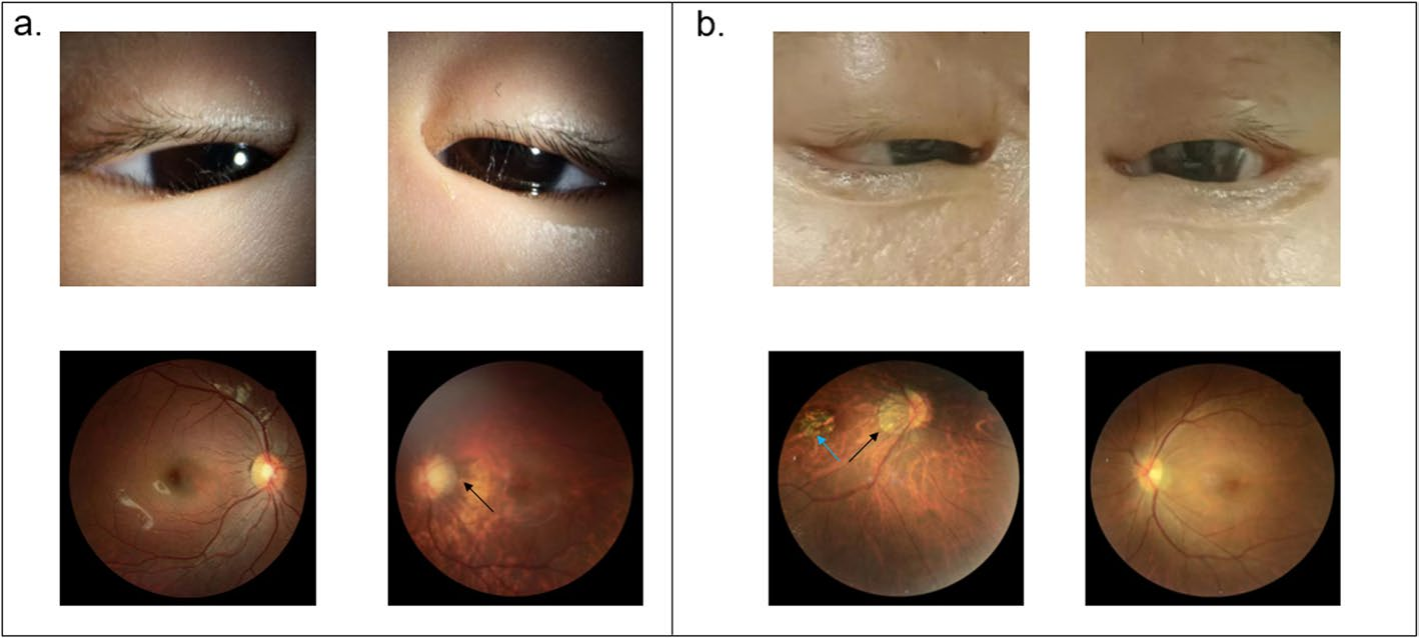
Clinical features of the BPES-diagnosed family members. **a** Both the proband and the mother show bilateral ptosis and epicanthus inversus. Black arrow refers to lacquer cracks, peripapillary atrophy. **b** Blue arrow indicates macular patchy chorioretinal atrophy. The black arrow also points to lacquer cracks, peripapillary atrophy

The other affected patient (I:2, Fig. 1a) of this family also demonstrated bilateral ptosis. The vertical diameter of the palpebral fissure was 3 mm (OD) and 5 mm (OS). Her axial length was 28.69 mm in the right eye and 26.30 mm in the left eye. Fundus examination indicated lacquer cracks, peripapillary atrophy, and macular patchy chorioretinal atrophy in the left eye but not in the right eye (Fig. 2b). Spherical equivalent was -15.00D (OD) and -7.88D (OS). Due to the poorly corrected vision of the right eye, this patient was also diagnosed with unilateral amblyopia.

### Molecular diagnosis

**Fig. 3:**
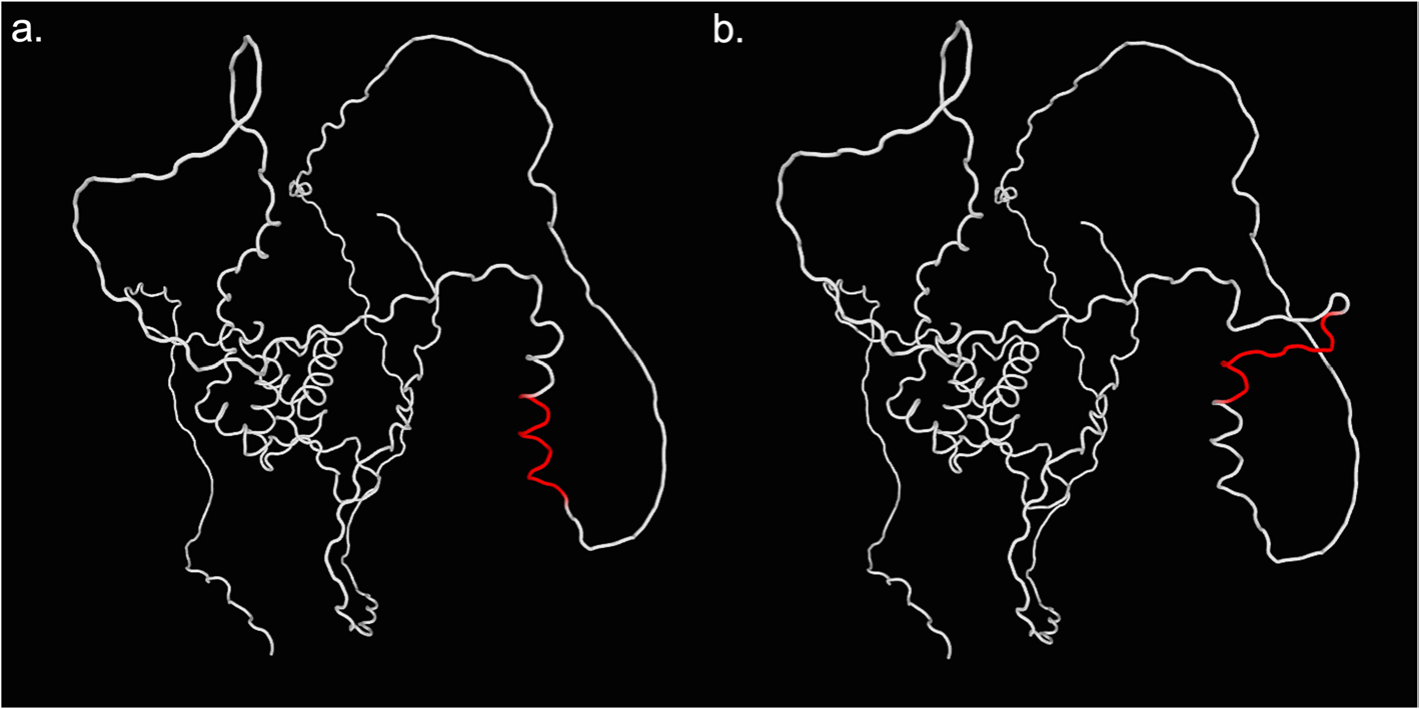
Protein structure of the wild-type and the variant c.672_701dup in *FOXL2. a* wild-type, the red region corresponds to the mutated region in (**b**); **b** c.672_701dup, the red region refers to the structural alteration of the mutated region

A heterozygous duplication variant c.672_701dup (p.A225_A234dup) in exon 1 of *FOXL2* was identified in the proband (II:1, Fig. 1b). Co-segregation analysis validated this disease causal variant in the proband’s mother (I:2, Fig. 1b), indicating an autosomal dominant inheritance pattern. Based on the guidelines of ACMG and AMP, the rare variant c.672_701dup, which had not been previously recorded in the ExAC, 1000G, orgnomAD databases, was estimated to be pathogenic moderate (PM2). Variant c.672_701dup was a 10 amino acids insertion that caused polyalanine expansion of the coding protein, but it did not lead to a frameshift variant. Thus, this variant was classified as pathogenic moderate (PM4). Disease-causing variant c.672_701dup had been previously reported in a Chinese BPES family with a de novo variant that was ranked as pathogenic moderate (PM6). Moreover, the ClinVar database assessed c.672_701dup as a pathogenic variant (P). Additionally, this variant may induce a structural alteration of the protein according to SWISS-MODEL prediction (Fig. 3). Therefore, the evidence suggested that this variant is the BPES-causing variant present in this family.

Combined with the clinical characteristics and molecular results, both the proband and his mother, who presented with anisometropia and unilateral PM, were diagnosed with type II BPES caused by the c.672_701dup variant in *FOXL2*. Additionally, the proband had congenital cataracts.

## Discussion

This study confirmed a non-frameshift variant c.672_701dup (p.A225_A234dup) in *FOXL2* as a disease-causing variant associated with a rare BPES pedigree that presents with anisometropia and unilateral PM. These results expand the phenotypic profile of *FOXL2* in BPES.

In addition to the four typical features of BPES – blepharophimosis, ptosis, epicanthus inversus, and telecanthus – this syndrome is most commonly characterized by microphthalmia and hyperopia. In a previous report, anisometropia and amblyopia were present in approximately 41% of patients, while only approximately 1% of patients presented with unilateral PM. However, none of the patients concurrently presented with unilateral PM, anisometropia, and amblyopia [10]. An additional study previously reported the disease causal variant c.672_701dup in a fourth-generation Chinese family, which included 13 patients. However, no manifestations of PM or congenital cataracts were recognized [15]. Moreover, previous research has also reported 20 patients with this specific variant in seven pedigrees, though none had PM or congenital cataracts [16]. Other studies have found that patients who carried this variant also had congenital hydronephrosis, ventricular septal heart defect (VSD), Duane syndrome, and/or growth hormone deficiency. Yet again, none had PM or congenital cataracts [17, 18]. We have reviewed the current studies that have reported different syndromes of BPES or ocular manifestations caused by variant c.672_701dup, which are presented in Table 1. Although more than half of the studies did not report the refractive status of the patients, about 41.7% (5/12) of the patients were diagnosed with isometropia, and 29.4% (5/17) of the patients had PM (Table 1). Furthermore, only one study reported congenital cataracts but did not genetically investigate the underlying cause (Table 1). Thus, this study expands the genotype–phenotype profile of the disease causal variant c.672_701dup in a BPES family with the rare manifestations of anisometropia, unilateral PM, and/or congenital cataracts. Additionally, the diverse clinical manifestations caused by this gene may be due to the variant's different types and positions and the distinct epigenetics of the same variant, which are valuable to investigate further.

**Table 1:** Reported Multiple Ocular Manifestations in BPES Caused by c.672_701dup or other disease causal variants in *FOXL2*

No	Variant	Anisometropia	PM	Congenital cataract	Other clinical features	Reference (PMID)
1	c.672_701dup	+	-	-	NA	31,048,069
2	c.672_701dup	-	-	NA	NA	17,968,144
	c.273C > G	-	-	NA	NA	
	c.663_692dup	-	-	-	NA	
	c.307C > T	+	-	-	NA	
	c.855_871dup	+	-	-	NA	
	c.576_577insC	+	-	-	NA	
3	c.672_701dup	NA	-	NA	NA	33,875,939
4	c.672_701dup	NA	NA	NA	NA	27,283,035
	c.663_692dup30	NA	+	NA	NA	
5	c.672_701dup	NA	NA	NA	NA	23,441,113
6	c.672_701dup	NA	NA	NA	NA	17,277,738
7	c.672_701dup	NA	NA	NA	NA	22,926,839
8	c.672_701dup	NA	NA	NA	NA	21,325,395
9	c.672_701dup	NA	NA	NA	NA	18,484,667
10	c.650C > G	NA	+ (2/3)	-	NA	22,312,189
11	c.844_860dup17	+ (1/4)	+ (2/4)	NA	NA	28,849,110
12	c.876dupC	-	-	-	NA	19,929,410
13	c.672_701dup	NA	NA	NA	Congenital hydronephrosis; hypertensive	25,192,944
14	c.672_701dup	NA	NA	NA	Duane syndrome	16,283,882
15	c.672_701dup	NA	NA	NA	2/3 skin syndactyly	18,642,388
	c.672_701dup	NA	NA	NA	Pediatric Burkitt lymphoma	
	c.672_701dup	NA	NA	NA	Small apical muscular ventricular septal heart defect	
16	NA	NA	NA	+	NA	35,219,116

“ + ” refers to positive result, “-” refers to negative result

*Abbreviations*: *BPES* Blepharophimosis, ptosis, and epicanthus inversus syndrome; *PM* Pathologic myopia, *NA* Not available in reported study

The Forkhead box L2 (FOXL2) gene (OMIM #605,597), which is a member of the highly conserved FOX superfamily, encodes a transcription factor that plays a role in the development of both the eyelids and ovaries [19]. The FOXL2 protein contains approximately 100 amino acids and is highly divergent in expression and function [19]. *FOXL2* was previously reported to be a candidate gene in the loci 3q22–q23 with translated truncated proteins identified in both types of BPES families [2]. It was then hypothesized that severe loss of function (LOF) variants in *FOXL2* lead to type I BPES, while type II BPES is caused by frameshift variants that result in elongation of the protein [20]. In the future, RNA isolation and quantitative polymerase chain reaction (qPCR) can be performed to further analyze the variant effect at the gene expression level and help to study the mechanism deeper. In this study, the inframeshift variant c.672_701dup causing 10aa elongation of products was discovered in a BPES family with rare ocular characteristics. The ovaries of the affected female were not dysfunctional, which cross-validated the above hypothesis. BPES requires eyelid surgery, particularly to correct ptosis, to allow for normal or improved visual development and cosmesis. In this study, the proband underwent ptosis and epicanthus surgery in both eyes and cataract surgery in the left eye during childhood. Proper interventional approaches at an early age are important for the adequate development of visual acuity and confidence in patients with BPES. Since BPES is an inherited disease commonly caused by disease causal variants in *FOXL2*, genetic screening early in life is a good tool to predict the type of BPES and to provide genetic information for clinicians to make an informed decision regarding therapeutic approaches.

However, there are some limitations of this study. This study involved only one family with two affected subjects, so more patients are warranted to replicate our findings. Although the reported gene was known, the newly discovered associated phenotypes need further functional study to investigate.

## Conclusions

In conclusion, this study revealed variant c.672_701dup in *FOXL2* as a BPES-causing variant in a family with the rare features of anisometropia, unilateral PM and/or congenital cataracts, thus expanding the phenotypic spectrum of *FOXL2*.

## Data Availability

The datasets generated and/or analysed during the current study are available from the corresponding author on reasonable request.
